# Experimental study of cavitating flow around a NACA 0012 hydrofoil in a slit channel

**DOI:** 10.1038/s41598-022-15256-w

**Published:** 2022-07-01

**Authors:** Sergey G. Skripkin, Mikhail A. Tsoy, Aleksandra Yu. Kravtsova

**Affiliations:** Kutateladze Institute of Thermophysics SB RAS, Novosibirsk, Russia 630090

**Keywords:** Energy science and technology, Engineering, Physics

## Abstract

An experimental study of a cavitating NACA0012 hydrofoil with aspect ratio 0.02 in a slit channel was carried out using a high-speed visualization at sampling rate more than 100 kHz. The features of the formation and development of cavities in a quasi-two-dimensional turbulent flow were studied. The most energetic modes of unsteady cavitation flow around the hydrofoil were obtained using dynamic mode decomposition. The presence of the second and third modes in the cavitation flow is shown here for the first time. It was shown that each of these modes corresponds to a certain period of development of the unsteady cavity. Experimental data on the reverse motion of the vapor–gas mixture into the inner region of supercavitation are presented.

## Introduction

A hydraulic microturbine equipment has been actively developed in recent years^1,2^. Microturbines are very mobile due to their small size and weight, which allows them to be used to generate electricity in hard-to-reach places, in pumping equipment, medical devices, and precision equipment. Since the operation of microturbines is largely autonomous, all key components of these systems must have a long life and be carefully designed. Cavitation is the main cause of breakdown in hydrodynamic systems^3–5^ and has scarcely been studied in small devices, when the distance between walls channel is of the order of 1–50 mm^6^.

Due to the dependence of the cavitation phenomenon on many factors, such as gas content, incoming flow velocity, and the surface morphology of body, the study of cavitation flows is carried out in well-controlled conditions on simple geometries: nozzles, cylinders, plates, and hydrofoils. It is possible to obtain the characteristics of the main flow and the cavitating body leading to a pressure drop and the formation of cavities.

The study of a cavitating body is basically carried out in tunnels with a three-dimensional turbulent flow, when the channel walls are located at a sufficient distance from each other. In this case, the cavitation near a two-dimensional hydrofoil in tunnels has a completely three-dimensional character due to side wall effects^7,8^. Arising strong lateral effects that can be observed both experimentally and numerically lead to the dependence of the flow and cavitation structures on the ratio of the tunnel depth, *d*, to its width, *w*^9^. The effect of decreasing the channel depth to critically small sizes, when the depth-to-width ratio is much less than unity (*d*/*w* << 1), on the occurrence of cavitation flows near the bluff bodies has not been practically studied. Therefore, experimental studies of cavitation flows in narrow slit channels are of decisive importance for the design of microturbine equipment.

In addition, the second main factor affecting the cavitation flow in microturbine equipment is the aspect ratio of a hydrofoil^10,11^. Holl and Wislicenus^12^ have found that the relationship between the initial cavitation number and the Reynolds number is different for hydrofoils with different chord lengths. They have shown that, in addition to the Reynolds number, the scale and velocity effects should also be considered in equipment design. Investigating cavitation flows over two different hydrofoils, Kuhn de Chizelle et al.^13^ and Luo et al.^14^ have each shown that decreasing the aspect ratio *s*/*C* by changing the chord length, *C*, enhances cavitation.

Kravtsova^15^ has performed particle image velocimetry (PIV) measurements near NACA0015 hydrofoils with different chord lengths for different cavitation numbers and angles of attack and have shown that, in the range from the hydrofoil leading edge to the 0.7*C* section, the profiles of the average flow velocity and turbulent pulsations of flow velocity differ slightly. However, near the trailing edge, there is a significant decrease in the maximum component of the average flow velocity for the larger hydrofoil, i.e., a hydrofoil with a smaller aspect ratio of 0.4, which leads to the occurrence of additional harmonics, whose presence has also been shown by Kawakami et al.^16^. Kawakami et al.^16^ have suggested that the presence of additional harmonics affects the hydrodynamics of cavitation flow and the fundamental shedding frequency of the cavity. It has been concluded that the influence of harmonics on the behavior of cavities should be given close attention when studying cavitation instability in small water tunnels.

Goncalves et al.^17^ were among the first to consider the change in the aspect ratio by changing the hydrofoil span. It has been shown that large vortex structures past a circular cylinder decrease significantly as the aspect ratio of the hydrofoil decreases. The minimum aspect ratio considered for a circular cylinder was 0.1. Large vortex structures formed around hydrofoils can generate in alternating forces in the transverse direction^18^. However, Gonçalves et al.^17^ have shown that no alternating forces are observed in the transverse direction for cylinders with an aspect ratio of less than 0.2. In this case, a quasi-two-dimensional turbulent flow results from the strong influence of near-wall flow friction. That is, a decrease in the aspect ratio leads to significant changes in the hydrodynamics of the cavitation flow.

Typically, NACA hydrofoils in a three-dimensional flow with an aspect ratio of more than 0.47 are studied. Incipient cavitation, sheet cavitation, cloud cavitation, and supercavitation are examined when the cavitation number changes from high to low by analyzing the flow structure of the high-speed visualization data. To simplify the analysis of simpler cavitation structures without losing the effect of the influence of the tilt angle, a low aspect ratio solution has been implemented, which allows high-speed imaging at high quality. We take into account two factors: the distance between the walls of the working channel and the aspect ratio of the cavitating body.

Thus, our work focuses the experimental studies of cavitation flows in a slit channel near a NACA0012 hydrofoil with a small aspect ratio (~ 0.02). The distance between the walls is 1.2 mm. To identify the features of cavitation flows in narrow slit channels, we compared the main characteristics of cavities for a larger aspect ratio with results obtained in previous studies. The main objective of the present study was to achieve a better understanding of the physical mechanisms governing cavitation processes at different scales.

## Experimental setup and measurement equipment

**Figure 1:**
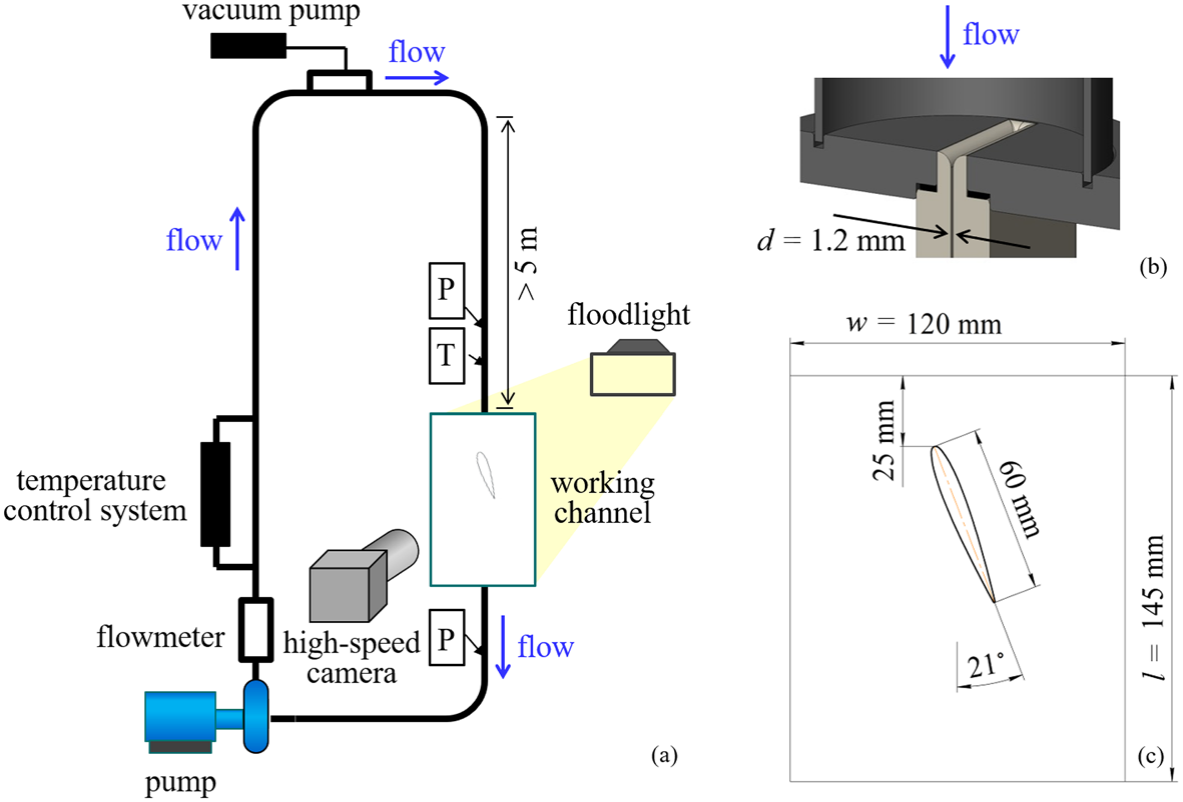
(**a**) Experimental loop. (**b**) Shape of the working channel inlet. (**c**) Scheme of the working channel.

Cavitation flow was studied in the loop hydrodynamic tunnel of the Institute of Thermophysics SB RAS, Novosibirsk (Fig. 1a). In the experiments, purified water containing no more than 7.3 mg/l of dissolved air was used. The amount of dissolved gas was minimized by iterative degassing, including flow turbulization at high Reynolds numbers, followed by the evacuation of released air bubbles from the reservoir at the top of the experimental setup using a vacuum pump. Liquid flow in the loop was produced by an EBARA 3LMH centrifugal pump with a power of 18.5 kW. The maximum fluid flow rate in the loop was 10 m^3^/h, which corresponded to an average flow velocity *U*_0_ = 20.5 m/s in the flow incident on the hydrofoil. The tunnel inlet was more than 50 diameters long to ensure that a fully developed turbulent flow entered the test section. A confusor (Fig. 1b) with a 7 mm tapering radius was placed in front of the test section to make the flow uniform at the inlet of the test section. The degree of flow turbulence at the inlet did not exceed 1%. The test section was a channel with a depth of *d* = 1.2 mm between two parallel transparent plates. The working channel was *l* = 145 mm long and *w* = 120 mm wide (Fig. 1c). The experimental body was a NACA0012 hydrofoil with a rounded trailing edge. The radius of the rounded trailing edge was 0.4 mm. The hydrofoil chord length, *C*, was 60 mm, and its span was 1.2 mm. The angle of attack of the hydrofoil relative to the main stream, *α*, was 21°. The aspect (span/chord) ratio (*AR*) of the hydrofoil was 0.02. The hydrofoil was made of plexiglas by milling. The test section was followed by a diffuser and a return tube that supplied liquid to the pump. A plate cooler was used to maintain a constant temperature. The liquid temperature was 27 °C. The experiments were carried out in the flow rate range from 5.3 to 8.4 m3/h. Starting from Q = 5.5 m3/h, cavitation was observed on the leading edge of the NACA foil. The Reynolds number was calculated as Re = *U*_0_ ∙ *C*/*ν*, where v is the kinematic viscosity, *U*_0_ = *Q*/*S* is the bulk velocity, where *Q* is the flow rate, and *S* is the cross-sectional area of the rectangular test section minus the hydrofoil midsection, and *C* is the hydrofoil chord length. The Reynolds number ranged from 7·10^5^ to 1.2·10^6^. Due to the peculiarities of the flow-pressure characteristics of the pump, and the high hydraulic resistance of the narrow gap of the working section, with an increasing flow rate, the pressure in the closed circuit also increased, which slowed down the decrease of cavitation number *σ* at high flow rates*.* The cavitation number *σ* = 2 (*P*_0_ − *P*_vap_)/*ρ* ∙ *U*_0_^2^, where *P*_0_ is the pressure at the inlet to the test section, *P*_vap_ is the vapor pressure, and ρ is the density of water. The cavitation number was varied from 3.2 to 2.3. To characterize the non-stationary process of the formation of cavities, we used the dimensionless Strouhal number (St). The Strouhal number was calculated as St = *f* ∙ *C*/*U*_0_, where f is the cavity shedding frequency, *C* is the hydrofoil chord length, and *U*_0_ is the average flow velocity.

Fast cavitation flows were captured using a high-speed Photron fastcam nova s12 camera with a sampling rate of more than 100 kHz. The size of the study area was 120 × 145 mm. The flow was illuminated with a floodlight using ground glass to uniformly scatter light from 20,000 lm built-in LEDs.

## Results

The results of the high-speed cavitation flow visualization in the slotted channel are described in “Cavitating NACA0012 hydrofoil: unsteady cavity” section and “Supercavitation NACA0012 hydrofoil: quasi-steady cavity” section. Two methods were used to create a cavitation flow in the working channel. The first method was quasi-stationary, where the flow rate in the working section increased slowly, and the time between studied points was about 20 min. The results of these measurements are given in “Cavitating NACA0012 hydrofoil: unsteady cavity” section. The second method of fluid acceleration was sharp, where the flow rate was increased from 0 to 6.8 m^3^/h in 10 s. The results of these measurements are given in “Supercavitation NACA0012 hydrofoil: quasi-steady cavity” section.

### Cavitating NACA0012 hydrofoil: unsteady cavity

**Figure 2:**
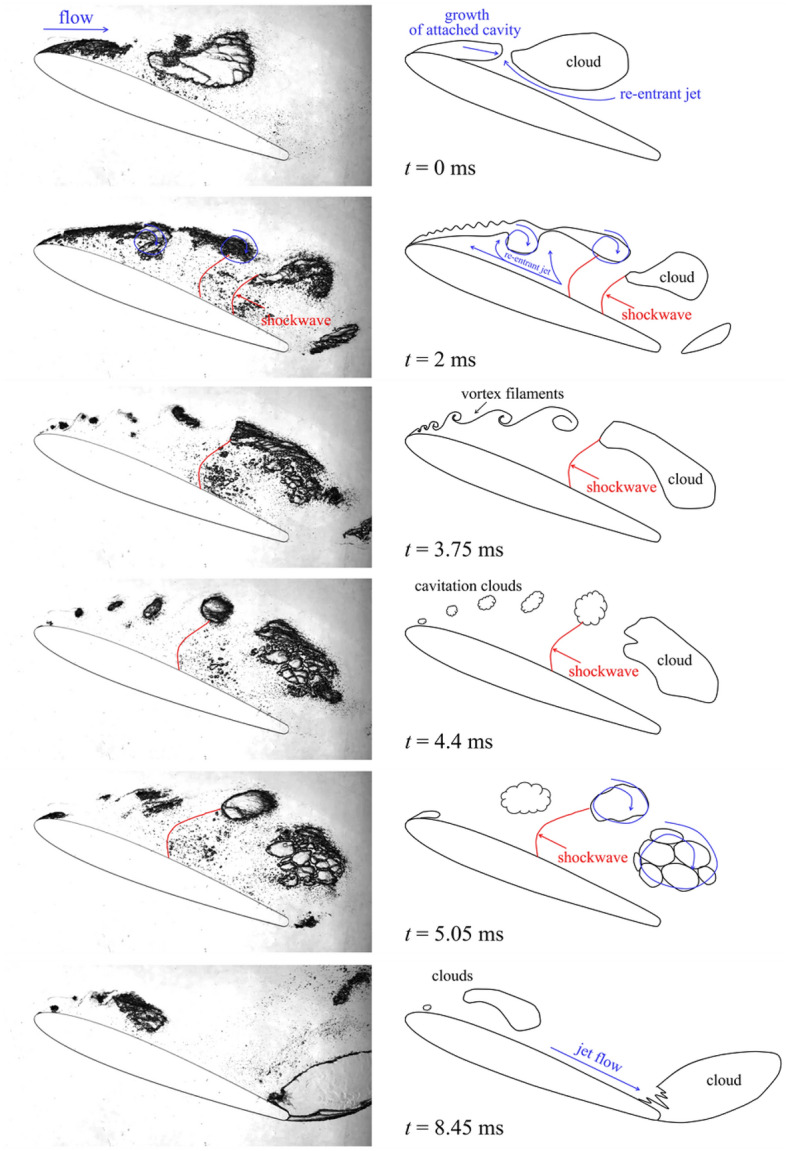
Cavitation cycle for the NACA0012 hydrofoil with *AR* = 0.02. *σ* = 2.41 (see Supplementary material).

The high-speed visualization revealed features of the formation and development of the cavitation flow past a NACA0012 hydrofoil with a small aspect ratio of 0.02. When the fluid was slowly accelerated in the experimental setup, an unsteady cavity was established near the body. The time evolution of the development and shedding of the unsteady cavity is shown in Fig. 2. The fluid flow was directed from left to right. The Reynolds number was 1.03·10^5^. The cavitation number was 2.41. A re-entrant jet developed under the cavity. The re-entrant jet propagated along the hydrofoil surface in the opposite direction to the main flow, as shown in the diagram. The detachment of the cavity first occurred in the rear of the cavity (Fig. 2, t = 0 ms) and then slightly upstream with the formation of separately rotating small cavitation regions. However, the attached cavity continuing to grow “absorbed” some of the detached clouds; the remaining cavitation clouds were carried downstream. As a result, the attached cavity of maximum length (Fig. 2, t = 2 ms) included a vapor–gas region, which was in contact with the hydrofoil surface and did not rotate relative to the hydrofoil and the detached cavity^19,20^, in which some of the small cavitation bubbles in the conglomerate rotated as a whole relative to each other and the upper hydrofoil surface. The maximum length of the attached cavity was more than 2/3*C* (Fig. 2, t = 2 ms). In the next stage of the development of the vapor–gas region after time t = 3.75, ms, the re-entrant jet reached the leading edge of the cavity and detached. The attached cavity broke up into several parts to form vortex filaments (Fig. 2, t = 3.75 ms), which is not typical for large angles of attack in hydrofoils with an aspect ratio of more than 0.417. However, for narrow slit channels, this type of breakup occurs regularly. The small spacing of ~ 1 mm between the walls of the test section prevented the development of large-scale vortex structures in the transverse direction and hence the growth of the cavity, resulting in partial breakdown rather than complete detachment of the cavity.

The destruction of thin vortex filaments at time t = 4.4 ms was followed by the formation of a sequence of small cavity clouds (Fig. 2, t = 4.4 ms).

The cavitation clouds detached from the hydrofoil surface were a conglomerate of flat vapor–gas bubbles. Vapor–gas cavities rotating and moving downstream as a whole could merge with each other and separate. When they merged, the thin liquid film at the interface disappeared, and a formation of ring waves on the lateral surface of the cavitation cloud was observed. As one vapor–gas flat cavity in the cavitation cloud grew, another adjacent cavity could decrease. When the cavitation cloud entered the high-pressure region, it began to collapse. Collapse always occurred only at one of the channel walls, which was due to the uneven pressure distribution on the wall of the slit channel.

A cavity also developed behind the rear rounded edge of the hydrofoil (Fig. 2, t = 8.45 ms). The beginning of its formation occurred in the antiphase with the cavity formed behind the leading edge of the hydrofoil, and its size could exceed half of the length of the hydrofoil. The cavity formed behind the trailing edge of the hydrofoil may have been inhomogeneous and consist of flat cavitation bubbles, which interact with each other during development. The separation of the cavity from the trailing edge of the hydrofoil was due to the motion of the liquid jet downstream along the hydrofoil surface, as shown in Fig. 2, t = 8.45 ms. The jet velocity exceeded the average flow velocity. The detached cloud was carried downstream and collapsed. When the liquid moved downstream along the hydrofoil surface, a new attached cavity began to form on the leading edge and the process repeated. The total time of the cavity growth and collapse cycle was T = 9.62 ms.

The development of cavities was greatly influenced by the pressure waves resulting from the collapse of cavitation clouds (Fig. 2). These pressure waves affected the size characteristics of the cavities. An attached cavity encountering a pressure wave in the flow increased in length and its growth slowed down for some time. In addition, it was found that, ahead of the pressure wave front, there was a collapse of detached cavitation clouds, and behind this front, there was a growth of cavitation bubbles which is not due to any other reasons.

### Supercavitation NACA0012 hydrofoil: quasi-steady cavity

**Figure 3:**
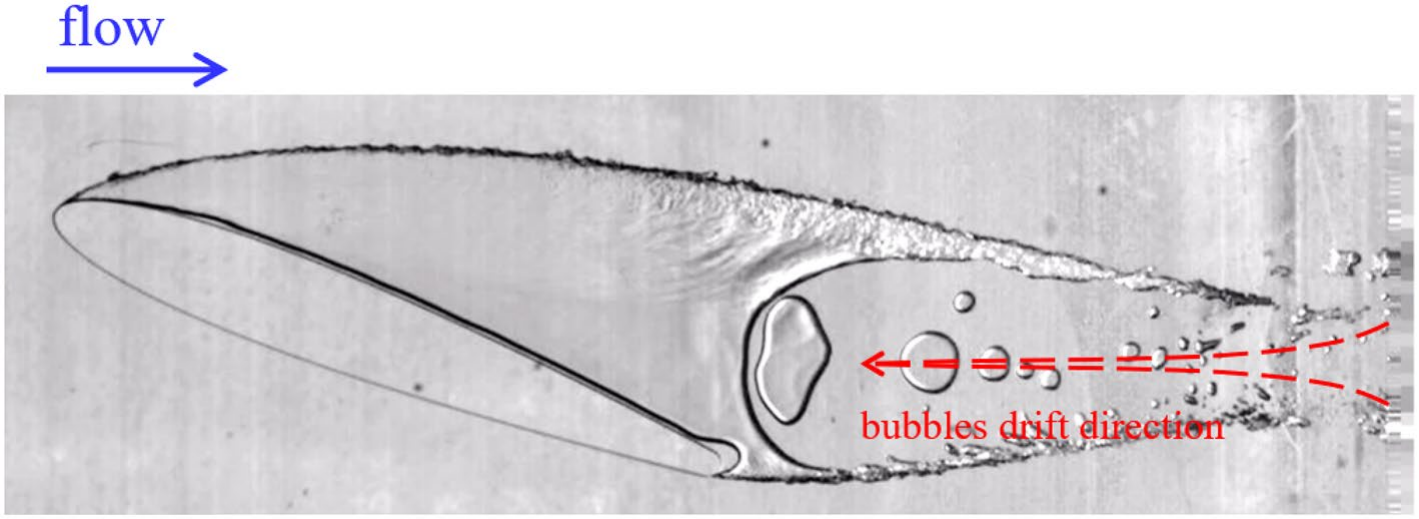
Supercavitating foil. A reverse motion of the water–vapor mixture phase occurred within the trailing edge of the cavity. Re = 9·10^5^. σ = 2.5 (see Supplementary material).

With a sharp increase in the flow rate from 0 to 6.8 m^3^/h, which corresponds to a Reynolds number of 8.9·10^5^, in contrast to the flow pattern shown in Fig. 2 (where the establishment of the mode was quasi-steady), the formation of a continuous supercavity was detected. The length of the cavity exceeded the length of the hydrofoil and ended already beyond the boundaries of the optically transparent test section (Fig. 3).

The cavitation number was *σ* = 2.5 (Re = 9·10^5^). Kelvin–Helmholtz instability developed at the upper boundary of the cavity that formed^21^; however, the wave structure formed at the upper boundary of the vapor–gas region also caused the formation of instability waves at the lateral boundary of the cavitation region. The visible front extended over a distance of 0.3*C*. Further, the waves were damped by friction against the channel walls.

Small vapor–gas regions moved opposite to the main flow into the inner region of supercavitation^22,23^, and their size increased. A reverse pressure gradient occurred in the cavity.

**Figure 4:**
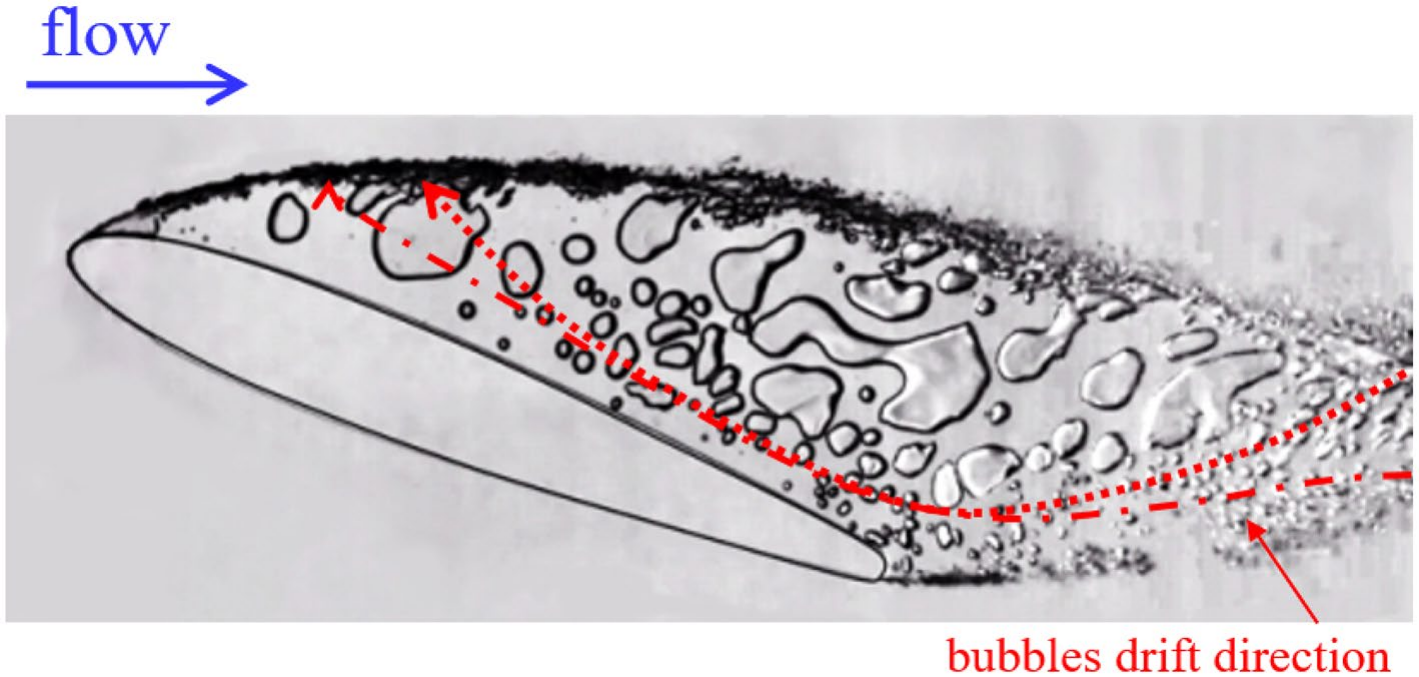
Supercavitating foil. A reverse motion to foil leading edge of the water–vapor mixture phase occurred within the cavity. Re = 1·10^6^. σ = 2.44.

With a decrease in the cavitation number, there was a more frequent exchange between the gas–vapor cavitation region and the main flow (Fig. 4).

Vapor–gas bubbles entering the supercavity propagated upstream and merged with the main part of the cavity. The motion of vapor–gas regions into the cavity occurred alternately from the upper and lower parts of the cavity. Figures 3 and 4 show the directions of reverse motion of vapor–gas regions for different cavitation numbers. The period of reverse motion obviously increased with a decreasing cavitation number, and there was a sharp change in the type of supercavitation region between the cavitation numbers of 2.5 and 2.44. In this case, the cavity became almost completely filled with water. This presumably led to an increase in pressure above the upper surface of the hydrofoil and hence to a decrease in the lift force of the hydrofoil, which is of great significance for hydrofoil applications.

## Analysis

Further analysis of the cavitation parameters was carried out for the unsteady cavity presented in “Cavitating NACA0012 hydrofoil: unsteady cavity” section. The re-entrant jet velocity was estimated from the high-speed visualization results. The re-entrant jet velocity was determined to be in the region of 0.65–0.75 *L*_max_. Markers for estimating the velocity were small-scale bubbles that, after the collapse of the main cavity, are entrained in the flow and carried upstream. The re-entrant jet velocity can be considered almost constant in this region and for about 43% of the cavity evolution period T. The re-entrant jet velocity was about one-third of the average flow velocity. These values are slightly lower than those obtained in the early studies of Pham et al.^24^ and Callenaere et al.^25^, and wall friction can be assumed to have a significant effect on the development of the re-entrant jet.

For all flow regimes considered, the velocities of the upstream propagation of pressure waves were estimated. For this, in the time series of digital images, we selected images of cavities with the largest volume and the greatest distance from the leading edge of the hydrofoil. After that, the time of collapse of the vortex cavity was fixed, and small individual cavitation bubbles in the region of the re-entrant jet above the hydrofoil were observed. As soon as the wave front reached this region, the simultaneous collapse of small cavities ahead of the front was recorded. The velocity was estimated from the time interval between frames using the scale factor. The accuracy of time estimation was about 1 frame, which, at a shooting frequency of 20 kHz and lengths of about 80 mm, gave an error within 10%. The propagation velocity varied between 350 and 400 m/s.

**Figure 5:**
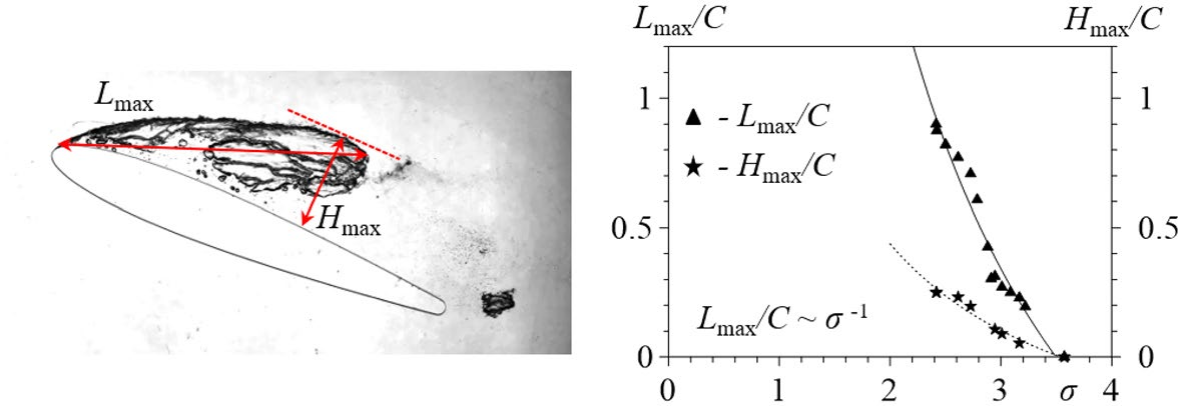
Characteristic attached cavitation parameters (left). Dependence of the maximum unsteady cavity length and the distance of the attached cavity from the hydrofoil on the cavitation number σ for the NACA0012 hydrofoil with the aspect ratio 0.02 (right).

One of the main characteristics of a cavity is its length^3,4,26,27^. The length, *L*_max_, of an unsteady cavity is defined as the distance from its origin to its trailing edge, as shown in Fig. 5. The distance of the cavitation cavity from the hydrofoil, *H*_max_, was determined as the normal distance from the hydrofoil surface to the maximally distant point of the vapor–gas region, as shown in Fig. 5. The cavity length increased with a decreasing cavitation number. The results of our measurements show that, for a well-streamlined smooth hydrofoil, the cavity length is related to the cavitation number as *L*_max_/*C* ~ *σ*^-1^. The obtained approximation relation confirms the hypothesis^28^ that, for symmetric hydrofoils, the exponent of the cavitation number, *σ*, depends primarily on the geometry of the hydrofoil.

For the investigated angle of attack, the propagation distance of the cavity did not exceed 25% of the hydrofoil chord length. As shown by Callenaere et al.^25^, the cavity thickness had a significant effect on the cavity instability induced by the development of a re-entrant jet in the rear of the cavity due to a high-pressure gradient^29^ and hence on the cavity shedding frequency^15,30^.

The shedding frequency of cavitation clouds is usually described by the dimensionless Strouhal number St.

To analyze the frequency parameters (characteristic harmonics) associated with the separation of the cavity from the top edge of the hydrofoil, we used image digital processing, which made it possible to identify and analyze cavitation clouds based on high-speed visualization data. In the first step, the contours of cavities were determined by the binarization method. The algorithm^31^ includes image conversion to grayscale, contrast optimization, noise removal, and multi-stage edge detection based on Otsu's double thresholding method. The next step was to fill the cavity pockets. The center-of-mass positions of cavitation cavities were determined. The main geometric and operating parameters were then obtained and analyzed. The volume of the gas–vapor cavity for each image can be calculated by multiplying the sum of pixels along the channel height by the pixel-to-millimeter conversion factor. The frequency characteristics of the cavitation flow were obtained by applying a fast Fourier transform (FFT) to the time evolution of the cavity volume for each of the modes studied. The pulsation spectra of the occurrence and collapse of cavitation clouds contained three regions with the highest PSD values: *f*_1_ ~ 100 Hz, *f*_2_ ~ 250 Hz and *f*_3_ ~ 550 Hz.

**Figure 6:**
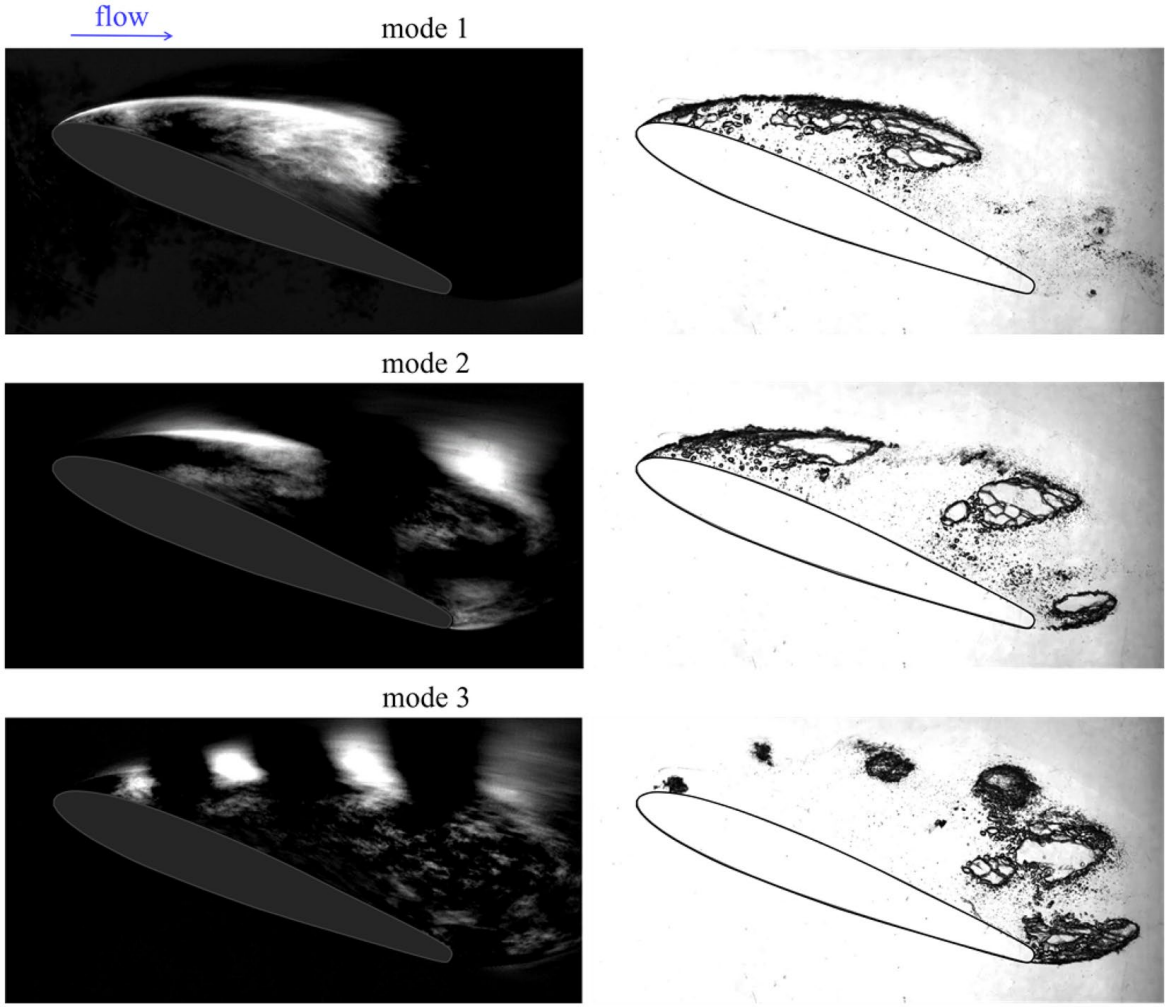
The first three most energetic modes of DMD analysis for cavitation flow around a NACA0012 hydrofoil. *σ* = 2.41.

To compare the frequency characteristics with cavitation flow patterns, we used dynamic mode decomposition (DMD) according to Higham et al.^32^ and Krake et al.^33^. Based on the high-speed visualization data of the cavitation flow around the NACA0012 hydrofoil in the slit channel, DMD also allowed us to identify the three most energetic modes with frequencies similar to those obtained by FFT. For a flow rate of 7.8 m^3^/h, the frequency of the fundamental mode associated with the formation of the largest cavitation cavities was 104 Hz, the second mode associated with the separation of individual smaller cavities had a frequency of 245 Hz, and the third mode associated with the frequency of the vortex train in the wake of the collapse of the cavity had a frequency of 558 Hz. The frequencies of higher modes depend on the number of modes used in dynamic decomposition and sometimes do not have much physical meaning. An example of the DMD decomposition and a comparison of each of these modes with the corresponding period of development of the unsteady cavity is shown in Fig. 6.

**Figure 7:**
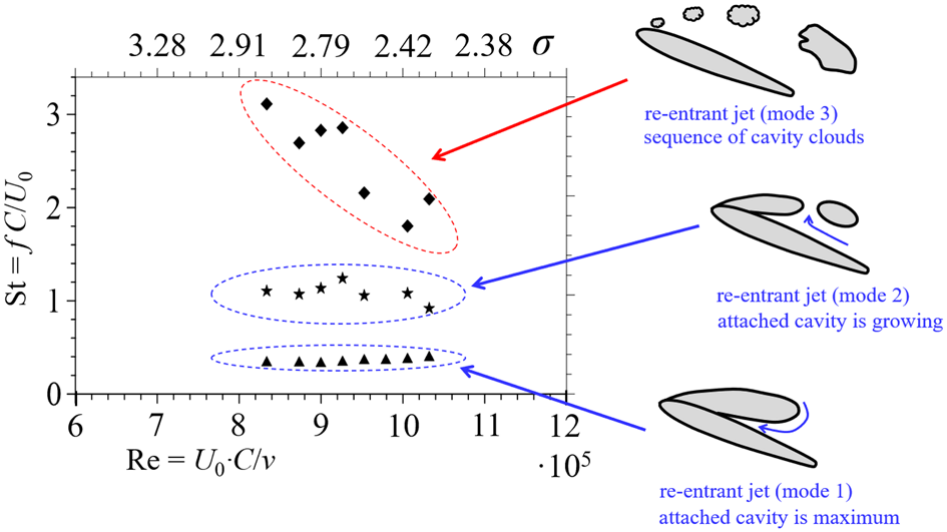
Calculated Strouhal numbers for different cavitation modes versus Reynolds numbers (bottom) cavitation numbers (top).

The main frequency of the unsteady cavity corresponding to the first mode is presented as the dimensionless frequency parameter, i.e., the Strouhal number, in Fig. 7. The Strouhal numbers obtained for the investigated NACA0012 hydrofoil with a very small aspect ratio were in the range from 0.3 to 0.4. As shown by Callenaere et al.^25^, Counter-Delgosha et al.^34^, Kravtsova et al.^28,30^, and Smith et al.^27^ for hydrofoils with a large aspect ratio, this range of Strouhal number corresponds to cloud cavitation. We observed this type of cavitation in all regimes of cavitation flow around the hydrofoil with a small aspect ratio. That is, when studying the cavitation flow around a hydrofoil, the size of the test section does not significantly affect the dimensionless parameter of the main frequency of pulsations of the cavity.

The presence of the second and third modes in the cavitation flow near two-dimensional hydrofoils was found here for the first time. A comparison of the second and third modes with a certain period of development of non-stationary cavities was carried out. The second mode corresponds to the separation of cavity described in “Cavitating NACA0012 hydrofoil: unsteady cavity” section, when the re-entrant jet propagating upstream separated part of the cavity at the trailing edge and was then captured by the growing attached cavity or carried downstream as a separate cavitation cloud (Fig. 6, Mode 2). The Strouhal number was around 1 (Fig. 7). The first and second modes are models and do not depend on the change in the cavitation number.

The third mode corresponds to the sequence of cavitation clouds formed by the train from the leading edge to the trailing edge of the hydrofoil (Fig. 6, Mode 3). This train of small cavities existed for about 0.25 T, and the dimensionless frequency of these clouds significantly depended on the cavitation number. That is, the Strouhal number increased with an increasing cavitation number. Smaller cavities moved faster, and their frequency was higher. The Strouhal number ranged from 2 to 3.

## Conclusions

Cavitation in a slit narrow channel was studied using a NACA0012 hydrofoil with an aspect ratio of 0.02 by means of high-speed visualization. Two different regimes of cavitation flow around the hydrofoil were recorded: unsteady and quasi-stationary, depending on the liquid time acceleration, from rest to reaching the cavitation regime. It was found that the cavitation cloud separated from the unsteady cavity consisted of a conglomerate of vapor–gas regions, which could move relative to each other and even merge to form larger cavities. Two types of surface instability of the cavity were identified—one due to Kelvin–Helmholtz instability and the other due to the merging of two vapor–gas regions. It was shown that the attached cavity could be conditionally divided into one part attached to the hydrofoil, which did not rotate relative to the hydrofoil, and another part detached from the surface, which consisted of separate parts rotating within the cavity. The separation of the cavity from the leading edge of the hydrofoil was accompanied by its separation into several parts due to the friction against the walls of the working channel.

Pressure waves caused by the collapse of gas–vapor cavities downstream were visualized, and their propagation velocity was estimated from the experimental data on the motion of the region of collapse/growth of small cavitation bubbles. The velocity of the re-entrant jet under the cavity was estimated. The most energetic modes of the cavitation flow around the hydrofoil were obtained using the DMD method. Each of these modes was shown to correspond to a certain period of development of the unsteady cavity. Experimental data were obtained on the reverse motion of the vapor–gas mixture into the inner region of supercavitation.

## Supplementary Information

Supplementary Information 1.

Supplementary Information 2.

## Data Availability

The datasets used and/or analyzed during the current study are available from the corresponding author on reasonable request.
