# Supplementary figures and images for: Experimental study of cavitating flow around a NACA 0012 hydrofoil in a slit channel
Source: Sci Rep. 2022 Jul 1;12:11182. doi: 10.1038/s41598-022-15256-w (PMC9249870; doi:10.1038/s41598-022-15256-w)

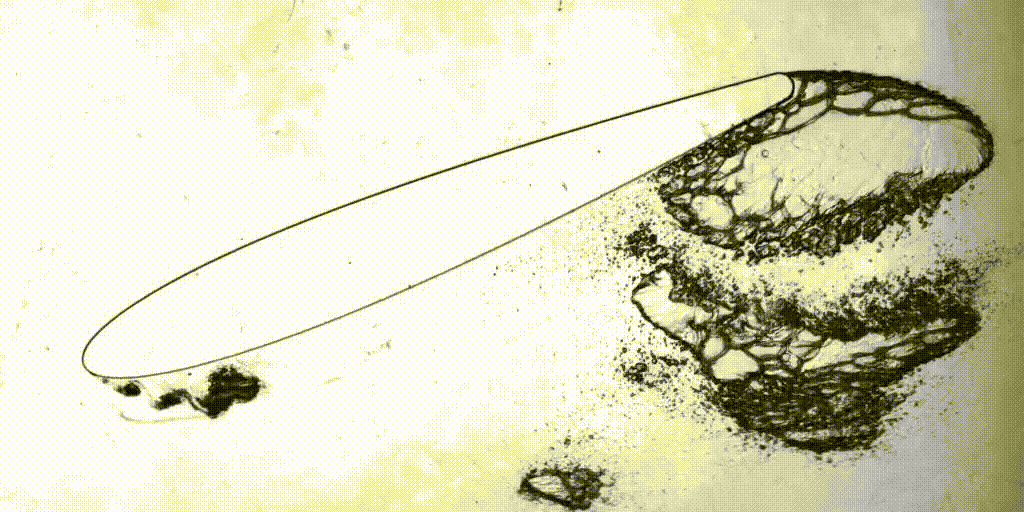

Supplement: Supplementary file 1 — Supplementary Information 1. [file 41598_2022_15256_MOESM1_ESM.gif]

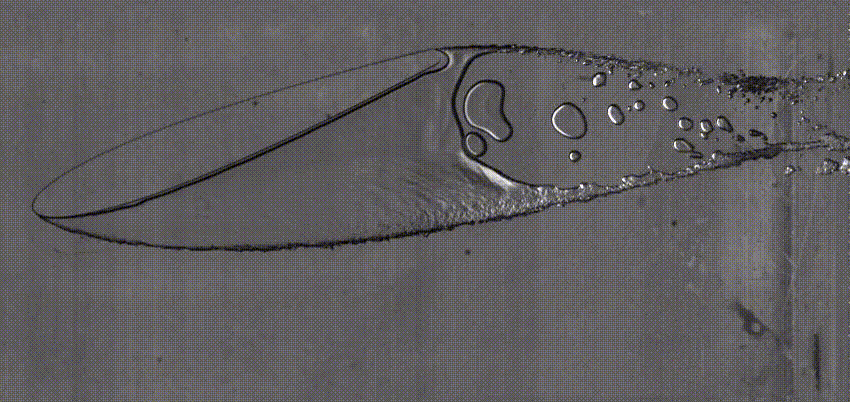

Supplement: Supplementary file 2 — Supplementary Information 2. [file 41598_2022_15256_MOESM2_ESM.gif]
